# Sex-related differences in the cortico-thalamo-cerebellar circuit in patients with schizophrenia

**DOI:** 10.3389/fpsyt.2026.1921553

**Published:** 2026-08-27

**Authors:** Youxue Zhang, Mingjun Duan, Dezhong Yao, Roberto Rodriguez-Labrada, Cheng Luo, Hui He

**Affiliations:** 1 The Clinical Hospital of Chengdu Brain Science Institute, MOE Key Lab for Neuroinformation, University of Electronic Science and Technology of China, Chengdu, China; 2 School of Education Science, Chengdu Normal University, Chengdu, China; 3 China-Cuba Belt and Road Joint Laboratory on Neurotechnology and Brain-Apparatus Communication, University of Electronic Science and Technology of China, Chengdu, China

**Keywords:** cortico-thalamo-cerebellar circuit, fMRI, functional connectivity, schizophrenia, sex differences

## Abstract

**Background:**

Schizophrenia is characterized by substantial clinical heterogeneity, with average differences between males and females in symptom presentation, cognitive function, and treatment response. The cortico-thalamo-cerebellar circuit (CTCC) has been implicated in schizophrenia; however, whether schizophrenia-related alterations in CTCC functional connectivity vary by sex remains unclear. This study examined diagnosis-by-sex interaction effects on CTCC-related functional connectivity using resting-state functional magnetic resonance imaging.

**Methods:**

Resting-state fMRI data were obtained from 108 patients with schizophrenia (53 females) and 93 healthy controls (54 females). Seed-based functional connectivity (FC) analysis and independent component analysis (ICA) were used to examine group-by-sex interaction effects on CTCC-related functional organization.

**Results:**

Significant diagnosis-by-sex interaction effects were identified in several connections involving CTCC-related basal ganglia, thalamic, cerebellar, and cortical regions. *Post hoc* analyses indicated that male patients exhibited increased FC between basal ganglia, thalamic, or cerebellar seed regions and temporal or calcarine cortical areas. Female patients showed a different pattern, including reduced FC between primary sensory cortical regions and basal ganglia or thalamic seed regions. These findings indicate that schizophrenia-related CTCC connectivity alterations differed between male and female participants. Chlorpromazine-equivalent dose was not significantly associated with the interaction-related functional connectivity values in either female or male patients.

**Conclusions:**

Sex moderated selected patterns of CTCC-related functional connectivity in schizophrenia. The findings represent group-level differences with substantial potential overlap between males and females and should not be interpreted as evidence of categorical brain sexual dimorphism or directional circuit mechanisms. Larger, prospectively sex-balanced studies incorporating longitudinal, dynamic, and effective-connectivity analyses are needed to confirm these findings and clarify their functional significance.

## 1 Introduction

Schizophrenia is a serious mental disorder that affects 24 million people in the world. It was defined as a serious mental illness characterized by delusions, hallucinations, disorganized behavior, and social withdrawal (1). Multiple clinical studies for individuals with schizophrenia reported that male patients typically present with more prominent negative symptoms whereas women exhibit more affective symptoms (2–4), and they showed evident differences in clinical therapeutic efficacy (5, 6). Despite sex differences in disease presentation have been widely reported, it remains unknown whether and how the effects of sex are reflected in the pathological mechanism of schizophrenia.

Sex-related variation in human brain structure and function remains an active and debated area of research. The investigations in morphology have revealed cerebral structure features differ between men and women, such as cerebral lateralization, and cortical thickness (3, 7, 8). A wide array of studies has proved that males and females have different patterns of connectivity in brain networks (9–11). Males typically show stronger inter-network functional connectivity (FC) whereas females have stronger intra-network FC. Recent studies have revealed significant differences in the functional connectivity between the basal ganglia and thalamus in men and women, as well as between these subcortical structures and the cerebral cortex and cerebellum (12, 13).

However, these findings should not be interpreted as evidence that human brains can be divided into two categorically distinct or sexually dimorphic types. Male and female distributions overlap substantially, and many reported regional differences are reduced after accounting for total brain size and other methodological factors. A comprehensive synthesis by Eliot et al. (14) concluded that few male–female brain differences are consistently reproduced beyond overall size-related effects. The authors also highlighted the susceptibility of neuroimaging studies, particularly fMRI studies, to limited statistical power, multiple-testing-related false-positive findings, population dependence, and poor replication. Conversely, Hirnstein and Hausmann (15) argued that rejecting a binary model of brain sexual dimorphism does not necessarily imply that all average sex-related differences are trivial, because even small effects may be relevant in particular developmental or clinical contexts. Therefore, sex-related neuroimaging findings should be understood as probabilistic group-level differences rather than categorical distinctions between male and female brains.

Previous studies have revealed that impairments in brain function differ between male and female patients with schizophrenia (16, 17). A research about magnetoencephalography showed that a widespread reduction in global functional connectivity was observed in chronic schizophrenia, with female patients showing the greatest deficit (17). Furthermore, male patients exhibit predominant dysfunction in the SMN, while female patients primarily show abnormalities in the DMN (16). As a complex mental illness, schizophrenia is often referred to as a ‘dysconnection’ syndrome whose underlying neurobiology stems from the failure of multiple brain regions to coordinate activity. Multiple studies have strongly supported the view that schizophrenia is characterized by extensive dysconnection in cortico-thalamo-cerebellar circuit (CTCC) (18–22). Patients with schizophrenia exhibit abnormal patterns of connectivity between the cerebellum and cortical/subcortical networks connected via CTCC (18). Moreover, the dysfunction of FC between the thalamus with the cerebellum and basal ganglia was also found in schizophrenia (19). The altered FC of the CTCC in schizophrenia patients indicated the impaired interaction among the cerebellum, the thalamus, the basal ganglia and the cortex in schizophrenia and its effects on cognitive processes. The sex difference has yet to be verified although abnormalities in CTCC have been widely reported in schizophrenia.

Over the past few decades, functional magnetic resonance imaging (fMRI) has become an effective tool for investigating *in vivo* brain function. It has been widely used to characterize functional responses, connectivity patterns, and the neural mechanisms underlying schizophrenia (23–26). Therefore, resting-state fMRI provides a useful approach for examining sex-related differences in the neural circuitry of schizophrenia. In the current study, we hypothesized that sex may influence the pathological alterations of the CTCC in schizophrenia. To examine this hypothesis at complementary levels, we combined seed-based functional connectivity analysis with independent component analysis (ICA). Seed-based FC is a hypothesis-driven approach that enables targeted examination of the connectivity between predefined key regions within the CTCC and the rest of the brain. Specifically, we assessed the whole-brain functional connectivity patterns of selected CTCC regions of interest. In contrast, ICA is a data-driven approach that identifies large-scale intrinsic functional networks without requiring *a priori* selection of seed regions. This analysis allowed us to evaluate sex-related alterations in connectivity within and between distributed brain networks associated with the CTCC. Thus, the seed-based analysis provided region- and circuit-specific information, whereas ICA provided a broader network-level characterization of functional dysconnectivity. By integrating these two complementary approaches, we aimed to obtain a more comprehensive understanding of sex-specific CTCC alterations in schizophrenia.

## 2 Methods

### 2.1 Participants

A cohort of 201subjects was used, 108 (53 females) schizophrenia patients and 93 (54 females) healthy adult controls who matched in age, and education. All of the schizophrenia patients were recruited from in-patient and out-patient treatment facilities in the Clinical Hospital of Chengdu Brain Science Institute and were diagnosed according to the Diagnostic and Statistical Manual of Mental Disorder, Fourth Edition (DSM-IV) by experienced psychiatrists. The Positive and Negative Symptom Scale (PANSS) was used to evaluate the severity of the patient’s condition. Two male and female patients lacked PANSS scales. All participants were right-handed and met the following inclusion criteria: 1) no history of substance abuse or dependence, 2) no history of significant systemic illness, head injury, or neurologic illness, and 3) no contraindications to MR scanning. None of the healthy controls (HC) had a history of mental illness or a family history of mental illness. All procedures performed in study involving participants were in accordance with the Ethics Committee of the Chengdu Mental Health Center in accordance with the Helsinki Declaration.

### 2.2 MRI acquisition

All MRI data was collected at a 3T GE DISCOVERY MR750 MRI scanner. During scanning, a foam head pad was used for head fixation and ear plugs were for noise abatement. The resting-state fMRI data were obtained using gradient-echo echo-planar imaging sequences, with an eight-channel phased array head coil. The parameters are as follows: TR = 2000 ms, echo time (TE) = 30 ms, flip angle (FA) = 90°, matrix = 64 × 64, field of view (FOV) = 240 × 240 mm^2^, slice thickness/gap = 4 mm/0.4 mm, number of slices = 34. All subjects are required to close their eyes during resting state scanning. Each scan lasted 510 s and generated 250 volumes. Subsequently, a three-dimensional fast spoiled gradient echo sequence was used to obtain the high-resolution T1-weighted images. The parameters are as follows: repetition time (TR) = 2300 ms, flip angle (FA) = 8°, matrix = 256 × 256, field of view (FOV)= 256 × 256 mm^2^, slice thickness = 1 mm, no gap, 213 slices).

### 2.3 Data processing

The DPABI software (https://rfmri.org/DPABI) and Matlab scripts were used to preprocess resting-state MRI data. Before the data preprocessing, the first five volumes were discarded to ensure stability magnetization. Then, the remaining volumes were corrected for differences in slice acquisition time. By the realignment to correct head motion between volumes. All the subjects with maximum mean framewise displacement (mFD) >0.25 mm were excluded (27). After that, the fMRI data of each participant was normalized using a non-linear registration into the standard Montreal Neurological Institute (MNI) space. The normalized fMRI images were smoothed with an 8-mm full-width at half-maximum Gaussian kernel. Subsequently, white matter (WM), cerebrospinal fluid (CSF), linear drift, the global averaged signals, and 24-head-motion parameters were extracted and were regressed from the fMRI data of each subject. Finally, the data were temporally filtered using a 0.01–0.08 Hz band-pass filter.

In the current study, grey matter volume (GMV) was calculated to correct the difference in brain size between females and males for further analysis. All T1-weighted images were inspected for artifacts or other quality questions and then processed using the standard “recon_all” processing stream by Freesurfer software (http://surfer.nmr.mgh.harvard.edu/).

### 2.4 Seed-based resting-state FC

Based on previous research on schizophrenia, this study systematically selected 12 core brain regions characterized by pathological features in patients as seed regions to investigate sex differences of schizophrenia in the CTCC (19, 28). The regions of interest are as follows: bilateral thalamus, basal ganglia (bilateral pallidum), bilateral insula, visual cortex (bilateral calcarine gyrus), auditory cortex (bilateral superior temporal gyrus), and somatosensory cortex (bilateral postcentral gyrus). These regions of interest were defined as 6-mm-radius spheres centered on coordinates derived from previous CTCC-related studies. This literature-based seed definition was used to provide a focused and reproducible assessment of regional functional connectivity. These seeds were not designed to distinguish the connectivity profiles of individual regional subdivisions. Then, the preprocessed fMRI data was used to calculate the Pearson’s correlation between the time series of the regions of interest and other voxels of the whole brain for each participant. After that, a Fisher’s r-to-z transform was applied to increase normality.

### 2.5 Group independent component analysis

The characteristics of brain networks were assessed employing independent component analysis on smoothed fMRI data using the GIFT software (http://mialab.mrn.org/software/gift/). First, principal component analysis (PCA) was performed to reduce the data dimensions. Subsequently, the independent group components were estimated by the Infomax algorithms. To determine the algorithmic reliability or stability, the ICASSO algorithm was set to rerun ICA 20 times. Next, Subject-specific spatial maps and time courses were then reconstructed from the group-level components and the original data using the spatiotemporal regression procedure.

The group data were decomposed into 53 independent components (IC), each of which was characterized by a spatial map and an associated time course. The spatial map represents the voxel-wise contribution of a coherent brain activity pattern, whereas the time course reflects the temporal fluctuations of that component. The component time courses were band-pass filtered at 0.01–0.08 Hz and converted to Z scores. Then, the 53 ICs were visually inspected according to established criteria for distinguishing neurobiologically meaningful resting-state networks from artifactual components. Components were retained when: (1) their peak clusters were predominantly located in gray matter; (2) they showed minimal overlap with white matter, cerebrospinal fluid, ventricles, large blood vessels, and brain boundaries; (3) their spatial distributions were anatomically meaningful and corresponded to previously established resting-state networks; and (4) their time courses were dominated by low-frequency fluctuations rather than high-frequency physiological or scanner-related noise. Components primarily reflecting head motion, vascular signals, cerebrospinal fluid fluctuations, susceptibility artifacts, or other non-neuronal sources were excluded. Based on these predefined spatial and temporal criteria, 13 components representing resting-state networks relevant to the cortico-thalamo-cerebellar circuit were retained for subsequent analyses. Component selection was based on their neuroanatomical and temporal characteristics rather than on diagnostic, sex, or group-by-sex statistical effects.

To explore the sex difference of the intra-networks FC, the spatial maps were gathered in each group in the first step. Next, the group comparison was restricted to the corresponding network mask which was created by the regions of the RSN in schizophrenia patients and HC, using the results of one-sample t-test, a significant level of p < 0.01, corrected by multiple comparisons via the false discovery rate correction.

### 2.6 Statistic analysis

Two-way analysis of variance (ANOVA) was employed to compare age, years of education, and mFD between four groups. For other demographic and clinical variables, differences between female and male patients with schizophrenia were compared using independent-samples t-tests. One-sample t-test was performed to show the strength of FC between seed points and other voxels across the brain in the four groups. Then, two-way ANOVA was also applied for comparison of the group * sex interaction and the main effects of group and sex on the map of the resting state FC based on seeds and ICs respectively. The Gaussian Random Field correction (GRF) (voxel-level p < 0.001, cluster level p < 0.01) for multiple comparisons was performed for the cluster-level analyses. We regressed age, years of education, mFD, and GMV. After that, a two-sample t-test was used for *post-hoc* tests. Finally, partial correlations were calculated between functional values in regions showing significant group*sex interaction and measures of disease severity (PANSS positive, negative, and total scores). Age, years of education, total intracranial volume, and mean framewise displacement (mFD) were included as covariates in the partial correlation analyses.

### 2.7 Validation analysis

To evaluate the potential influence of antipsychotic treatment on functional connectivity. Chlorpromazine-equivalent doses were compared between female and male patients with schizophrenia. Within two patient groups, partial correlation analyses were further performed between chlorpromazine-equivalent dose and functional connectivity values extracted from regions showing significant group*sex interaction effects, controlling for age, years of education, total intracranial volume, and mean framewise displacement.

## 3 Results

### 3.1 Demographic and clinical characteristics

The schizophrenia group included 53 female and 55 male patients, whereas the healthy control group included 54 female and 39 male participants. Pearson’s chi-square test showed no statistically significant difference in sex distribution between schizophrenia and healthy groups (χ^2^ = 1.6224, p = 0.2028). Age, years of education, and mFD did not differ among the four groups by ANOVA. Moreover, there are no differences in the level of duration of illness and clinical characteristics in schizophrenia patients. Details of demographic characteristics of groups are showed in **Table 1**.

**Table 1:** Demographic and clinical characteristics of participants.

Demographic variables and clinical characteristics	SZF (n=53)	SZM (n=55)	HCF (n=54)	HCM (n=39)	T/F	p
Age(year)	35.36(13.10)	36.38(12.70)	37.17(13.97)	35.90(13.43)	0.18	0.91^a^
Education(year)	13.04(2.96)	12.46(3.31)	12.22(4.36)	13.59(3.48)	1.35	0.26^a^
mFD(mm)	0.12(0.05)	0.15 (0.09)	0.14 (0.05)	0.14(0.05)	2.61	0.052^a^
Chlorpromazine Equivalent Dose(mg/day)	218.79(210.87)	269.90(196.28)	–	–	1.29	0.12^b^
Duration of illness(year)	8.82(8.97)	11.70(10.31)	–	–	1.52	0.13^b^
PANSS						
Total scores	70.00 (17.14)	64.56(18.78)	–	–	1.57	0.12^b^
Positive scores	18.02 (5.28)	16.29 (6.65)	–	–	1.49	0.14^b^
Negative scores	20.19 (8.40)	17.47 (8.46)	–	–	1.67	0.10^b^
General scores	31.79 (6.67)	30.80 (8.07)	–	–	0.70	0.49^b^

Demographic data are shown as mean (standard deviation).

^a^ The P value of the two-way ANOVA.

^b^ The P value of the two-sample t-test.

SZF, females with schizophrenia; SZM, males with schizophrenia; HCF, female healthy controls; HCM, male healthy controls; mFD, mean framewise displacement; PANSS, Positive and Negative Syndrome Scale..

### 3.2 Sex-specific alterations in seed-based functional connectivity

**Figure 1:**
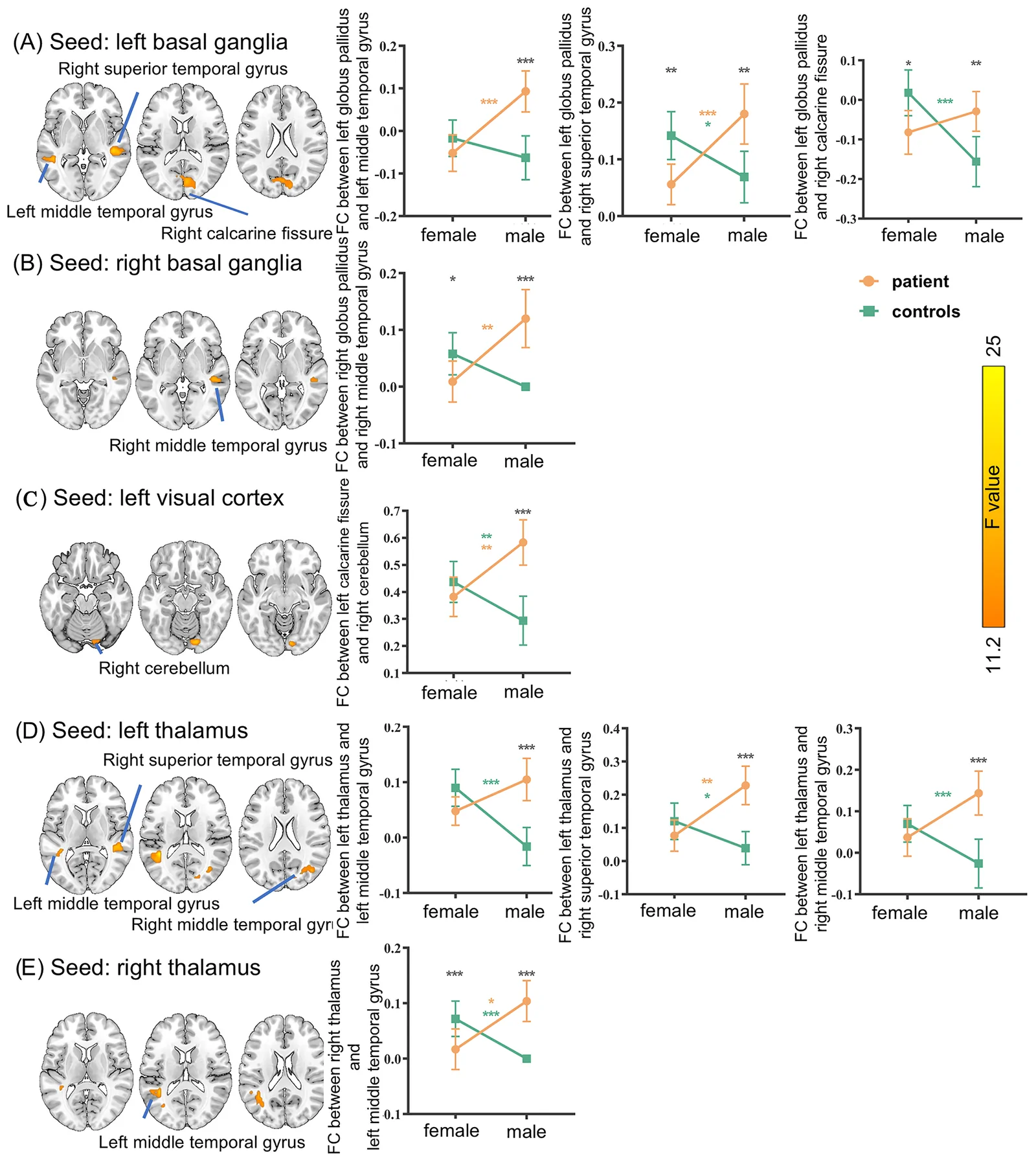
Seed-based functional connectivity analyses were performed to investigate sex-related alterations within the cortico-thalamo-cerebellar circuit (CTCC). Brain regions showing significant group * sex interaction effects are presented for each seed region, including **(A)** the left basal ganglia, **(B)** the right basal ganglia, **(C)** the left visual cortex, **(D)** the left thalamus, and **(E)** the right thalamus. Brain maps illustrate the anatomical locations of regions showing significant functional connectivity alterations, and line plots display connectivity differences between female and male patients with schizophrenia and healthy controls. The orange lines represent patients with schizophrenia, and the green lines represent healthy controls. The x-axis indicates sex (female and male), and the y-axis represents functional connectivity strength between the seed regions and target regions. Significant group × sex interaction effects indicate distinct sex-dependent connectivity patterns within the CTCC. Statistical significance is indicated by asterisks (*p < 0.05, **p < 0.01, ***p < 0.001). The color bar represents the F value of the interaction effects.

Our findings demonstrate significant main effects of both group and sex on functional connectivity throughout the CTCC pathway, encompassing multiple regions including the cerebellum, thalamus, basal ganglia, and cerebral cortex. For details, see **Supplemental Materials**. As shown in **Figure 1**, the ANOVA results revealed significant group-by-sex interaction effects in multiple brain regions. Compared to male patients and healthy female subjects, female patients exhibited decreased FC between the left globus pallidus and the right superior temporal gyrus, between the right globus pallidus and the right middle temporal gyrus, and between the right thalamus and the left middle temporal gyrus (p < 0.01, GRF-corrected). Additionally, compared to healthy female subjects, female patients showed significantly reduced FC strength between the left globus pallidus and the right calcarine cortex (p < 0.01, GRF corrected).

Furthermore, comparative analysis revealed that male patients exhibited increased functional connectivity across specific brain regions relative to both female patients and healthy male controls (p < 0.01, GRF corrected). The enhanced connectivity was observed between the following region pairs: (1) the left globus pallidus and left middle temporal gyrus; (2) the left globus pallidus and right superior temporal gyrus; (3) the right globus pallidus and right middle temporal gyrus; (4) the left pericalcarine cortex and right cerebellum; (5) the left thalamus and right superior temporal gyrus; and (6) the right thalamus and left middle temporal gyrus. Compared to healthy male subjects, male patients demonstrated increased functional connectivity between the left globus pallidus and the right pericalcarine cortex, between the left thalamus and the left middle temporal gyrus, and between the left thalamus and the right middle temporal gyrus (p < 0.01, GRF corrected). No significant correlation result was observed between group * sex interaction effect and clinical score.

### 3.3 Sex-specific alteration of the intra-network functional connectivity

**Figure 2:**
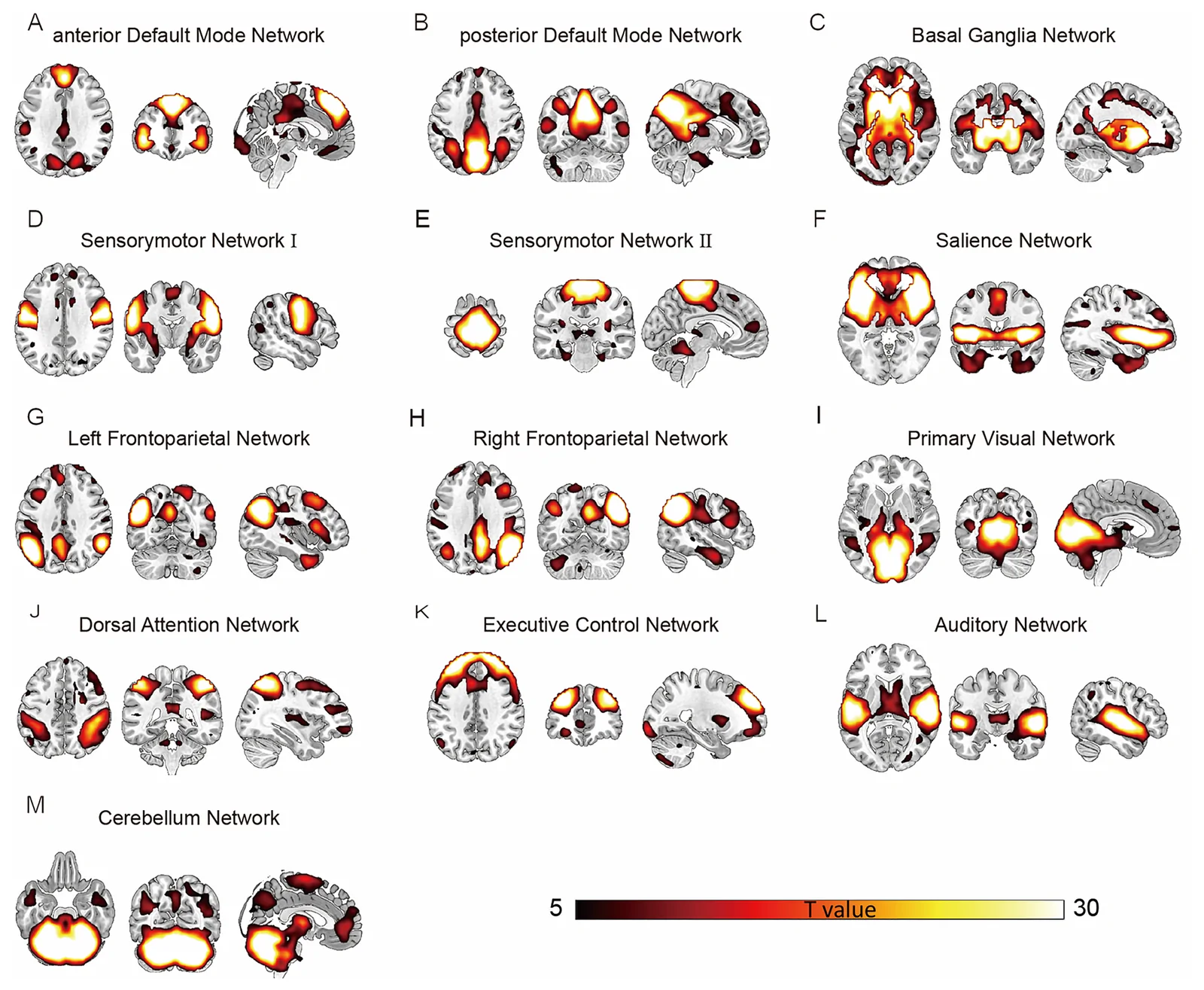
Spatial maps of identified functional networks from schizophrenia patients and healthy controls. (t contrast thresholded for positive values) **(A)** anterior default mode network, **(B)** posterior default mode network, **(C)** basal ganglia network, **(D)** somatosensory network I, **(E)** somatosensory network II, **(F)** salience network, **(G)** left frontoparietal network, **(H)** right frontoparietal network, **(I)** primary visual network, **(J)** dorsal attention network, **(K)** executive control network, **(L)** auditory network, **(M)** cerebellum network.

In total, group ICA resulted in 53 IC of which 13 components were chosen as networks of interest, including two somatosensory networks, primary visual network (VN), auditory network (AUN), anterior default mode network (aDMN), posterior default mode network (pDMN), executive control network (ECN), salience network (SN), dorsal attention network (DAN), left frontoparietal network (LFPN), right frontoparietal network (RFPN), cerebellar network (CN) and basal ganglia network (BGN) (**Figure 2**).

Analysis of intra-network functional connectivity revealed significant main effects of both sex and group on functional connectivity within several major networks, including the BGN, CN, SMN, and higher-order networks such as the default mode network (DMN). For details, see **Supplemental Materials**. No significant group * sex interaction effect was observed.

### 3.4 Validation result

Chlorpromazine-equivalent doses were comparable between female and male patients with schizophrenia, with no statistically significant difference (**Table 1**). To assess whether the interaction findings were associated with current antipsychotic treatment, correlation analyses were conducted separately in female and male patients. No significant associations were identified between chlorpromazine-equivalent dose and the functional connectivity values extracted from any region showing a significant group × sex interaction effect in either sex (all p > 0.05).

## 4 Discussion

Our study was aim to figure out whether and how the sex effects affect the pathological mechanism of schizophrenia patients in CTCC pathway. To this aim, we leverage the method of seed-based FC and ICA to explore the sex-specific alteration of this circuit in schizophrenia. We revealed distinct sexually dimorphic functional alterations within the CTCC pathway in schizophrenia. Specifically, female patients exhibited significantly weakened connectivity between the primary sensory cortex and the basal ganglia/thalamus, alongside abnormally enhanced connectivity and intrinsic basal ganglia networks. In contrast, male patients demonstrated hyperconnectivity between the primary motor/sensory cortices and the basal ganglia, thalamus, and cerebellum, but exhibited significantly reduced functional integration within the intrinsic basal ganglia networks.

Previous studies have confirmed that the basal ganglia play a crucial role in motor control and action selection (29, 30). Furthermore, it has been established that the basal ganglia, cerebellum, and cerebral cortex form an integrated, topographically organized network (30, 31). This network may support the integration of motor, cognitive, and emotional functioning through coordinated activity in the CTCC pathway. As a core pathological circuit in schizophrenia patients, the disruption of the CTCC can lead to cognitive impairment and dyscoordination of somatosensory and mental processes in schizophrenia (32, 33). Our findings extend these observations by identifying the FC of the CTCC. We observed female patients showed significantly decreased connectivity between the primary sensory cortex and the basal ganglia. This suggests abnormal activity of the basal ganglia of female patients in the CTCC pathway. Such activity may impair the basal ganglia’s inhibitory influence on the thalamus (34), leading to a overactivation of the CTCC. Concurrently, decreased functional connectivity was observed between the thalamus and the primary cortex. These findings suggest disrupted functional integration within the basal ganglia-thalamo-cortical pathway in female patients with schizophrenia, which may be associated with altered sensory and cognitive information processing. However, the directionality and underlying mechanisms of these connectivity changes cannot be inferred from the present resting-state data.

Male patients with schizophrenia exhibit distinct pathological connectivity patterns within the CTCC pathway: compared to female patients, they show significantly elevated functional connectivity strength between the basal ganglia and temporal lobe, between the basal ganglia and calcarine cortex, as well as between the thalamus and temporal lobe. As the relay station for sensory and motor information in the brain, the thalamus is responsible for transmitting information from the basal ganglia to the cerebral cortex. Abnormal activities between the basal ganglia and cerebral cortex in male patients with schizophrenia may lead to erroneous filtering by the thalamus in the CTCC pathway (35), resulting in the transmission of aberrant information to the cerebral cortex and subsequently causing clinical symptoms such as auditory and visual hallucinations. More notably, the abnormally enhanced connectivity was observed between the cerebellum and the calcarine cortex in male patients. Given the roles of the cerebellum and visual cortex in sensory integration processing, this finding may reflect the cerebellum may attempt to correct signal processing deviations in the basal ganglia-cortical circuit by enhancing the integration of erroneous motor prediction signals (36). However, this hyperconnectivity may instead exacerbate the dissociation between motor planning and execution, leading to more pronounced negative symptoms in male patients (37). Unlike the inhibitory phenotype observed in the primary cortex-basal ganglia connectivity of female patients, the pathological profile in male patients is characterized by a global hyperactivation across the hierarchical regulatory networks, specifically the “basal ganglia-thalamus-cerebellum” or “basal ganglia-thalamus-cortex” pathways.

The current study has several limitations. First, the sample sizes of the four subgroups were not fully balanced. Although the sex distribution did not differ significantly between the schizophrenia and healthy control groups (χ^2^ = 1.6224, p = 0.2028), the male healthy control subgroup was relatively small (n = 39) compared with the other three subgroups. Because the primary analyses focused on group*sex interactions, the smaller cell size may have reduced the statistical power and precision of the interaction estimates, particularly in the whole-brain voxel-wise analyses. Therefore, the present findings should be interpreted cautiously and require replication in larger, independently recruited, and prospectively sex-balanced samples. Future studies should also evaluate the robustness of these findings using matched-subsample or independent-cohort sensitivity analyses. Second, the biological mechanisms underlying the observed sex-related functional connectivity differences were not directly examined. In particular, the present study did not include endocrine, genetic, immune, or neurochemical measurements. Therefore, the imaging findings should not be interpreted as evidence for any specific biological mechanism. Future studies incorporating direct biological measurements are needed to clarify the mechanisms underlying sex-related CTCC alterations in schizophrenia. Third, the present study primarily focused on the CTCC. Because schizophrenia involves abnormalities across multiple large-scale brain systems, future studies integrating multiple functional networks and multimodal neuroimaging measures may provide a more comprehensive understanding of sex-related pathophysiological mechanisms. Finally, static resting-state functional connectivity measures statistical coupling between brain regions but cannot determine the direction of information flow or establish causal interactions within the CTCC. Future studies combining dynamic analyses with effective-connectivity methods, particularly dynamic causal modeling, are needed to investigate directed interactions among cortical, thalamic, basal ganglia, and cerebellar regions. Granger-causality-based analyses may provide additional information regarding temporal precedence, although their application to fMRI requires cautious interpretation because of regional variation in the hemodynamic response. Longitudinal and task-based designs may further clarify how sex influences CTCC dynamics across the course of schizophrenia.

## 5 Conclusions

In conclusion, this study illustrates that the pathological mechanism of schizophrenia in CTCC differs between male and female patients. We observed males with schizophrenia showed disruption activity in the primary cortex, while females with schizophrenia showed altered FC in the entire CTCC pathway. Such sex-specific alteration in this circuit may reflect on the sex differences of the clinical symptoms, and provide new insights into treatment methods and therapeutic efficiency of schizophrenia.

## Data Availability

The original contributions presented in the study are included in the article/**Supplementary Material**. Further inquiries can be directed to the corresponding author/s.
