# Supplementary material for: Sex-related differences in the cortico-thalamo-cerebellar circuit in patients with schizophrenia
Source: Front Psychiatry. 2026 Aug 27;17:1921553. doi: 10.3389/fpsyt.2026.1921553 (PMC13558228; doi:10.3389/fpsyt.2026.1921553)
Supplement: Supplementary file 1 [file DataSheet1.pdf]

Supplementary Results

S1. Compared with healthy controls, patients with schizophrenia showed significantly increased functional connectivity between the auditory cortex and the cerebellum, inferior parietal angular gyrus, thalamus, sensorimotor area, and precuneus; between the basal ganglia and the middle temporal gyrus and hippocampus; between the insula and the temporal lobe, postcentral gyrus, and medial superior frontal gyrus; between the sensorimotor cortex and the parietal lobe, cerebellum, and thalamus; between the visual cortex and the cerebellum, middle frontal gyrus, and thalamus; as well as between the thalamus and the sensorimotor cortex, occipital lobe, lingual gyrus, and parahippocampal gyrus. ($p < 0.01$, GRF-corrected)

In contrast, patients exhibited significantly decreased functional connectivity between the auditory cortex and the postcentral gyrus and lingual gyrus; between the basal ganglia and the cerebellum, cingulate gyrus, insula, precentral gyrus, thalamus, sensorimotor area, and supramarginal gyrus; between the insula and the cerebellum, globus pallidus, caudate nucleus, sensorimotor area, parietal lobe, and frontal lobe; between the visual cortex and the postcentral gyrus and occipital lobe; within regions of the sensorimotor cortex ; between the thalamus and the cerebellum, sensorimotor area, frontal lobe, insula, and parietal lobe; and within regions of the thalamus itself. ($p < 0.01$, GRF-corrected)

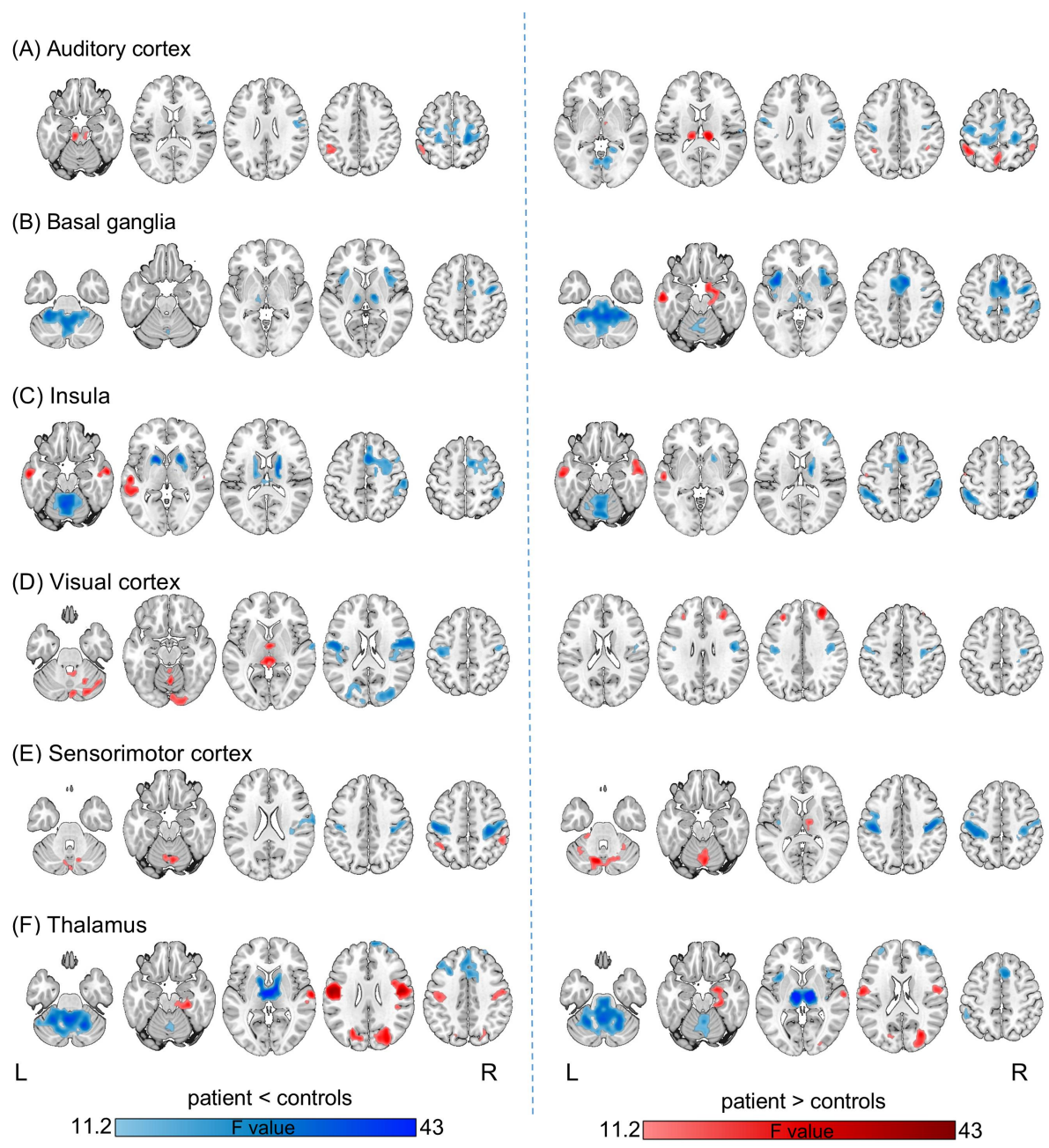

Figure S1. Significant main effect of group on seed-based functional connectivity. (voxel-level $p < 0.001$, cluster-level $p < 0.01$, gaussian random field corrected for multiple comparisons) $*p < 0.05$; $**p < 0.01$; $***p < 0.001$

S2. Compared with males, females exhibited significantly higher functional connectivity strength between several brain regions, including the auditory cortex and the occipital lobe, temporal lobe, and supramarginal gyrus; the basal ganglia and the occipital lobe, cuneus, and cerebellum; the insula and the cerebellum, cuneus, fusiform gyrus, and superior parietal angular gyrus; within the visual cortex; as well as between the thalamus and the inferior temporal gyrus. ($p < 0.01$, GRF-corrected)

Conversely, females showed significantly lower functional connectivity strength compared to males in pathways including the auditory cortex and the putamen, globus pallidus, and inferior parietal angular gyrus; the basal ganglia and the inferior temporal gyrus; the visual cortex and the parahippocampal gyrus and temporal lobe; and between the sensorimotor cortex and the medial superior frontal gyrus ($p < 0.01$, GRF-corrected).

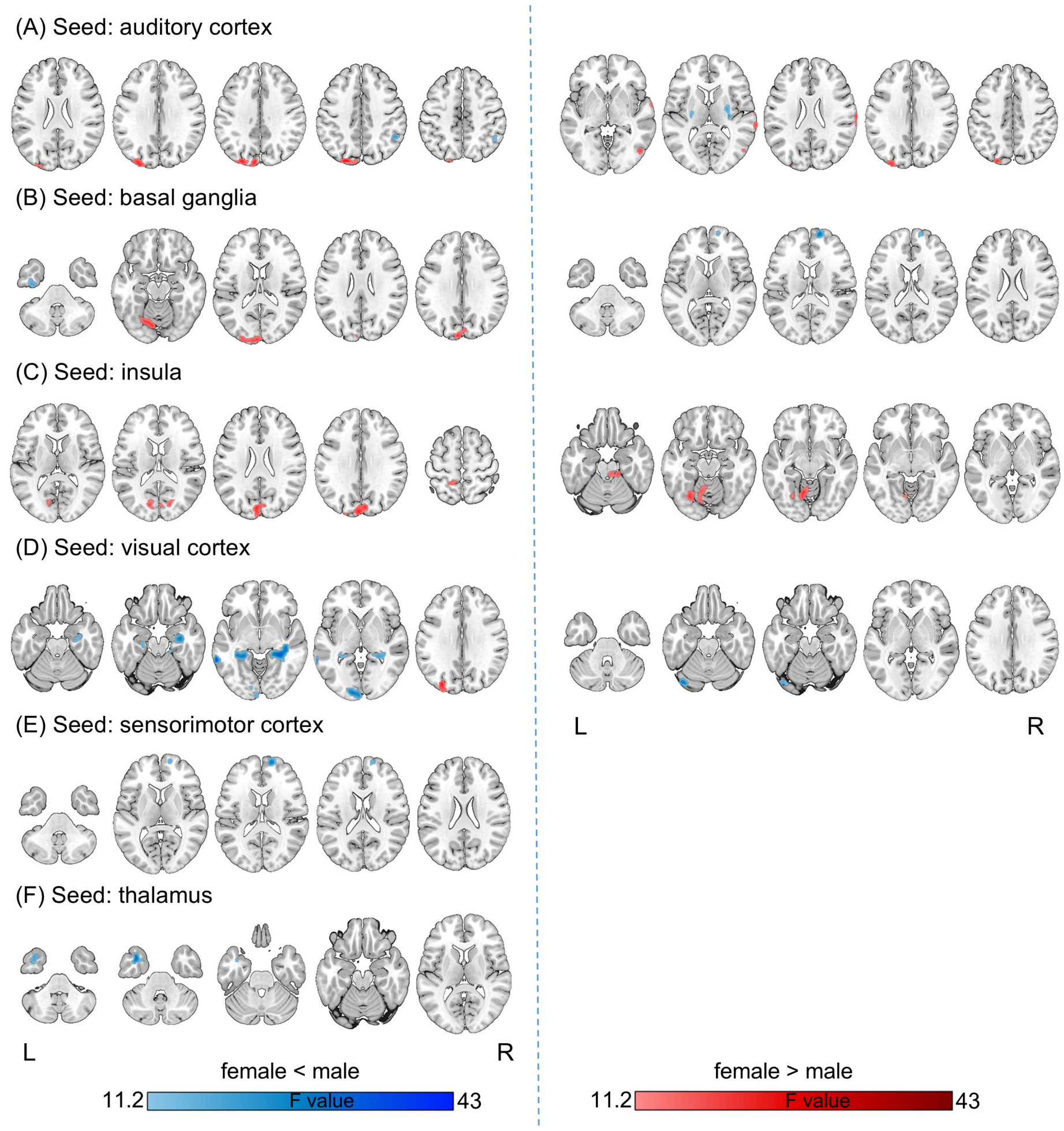

Figure S2. Significant main effect of sex on seed-based functional connectivity. (voxel-level $p < 0.001$, cluster-level $p < 0.01$, gaussian random field corrected for multiple comparisons) *$p < 0.05$; **$p < 0.01$; ***$p < 0.001$

S3. Based on the main effect of group from the ANOVA results, patients with schizophrenia showed significantly increased functional connectivity within multiple resting-state networks compared to healthy subjects, including the anterior default mode network (right orbital inferior frontal gyrus), the basal ganglia network (right caudate nucleus and left globus pallidus), the cerebellar network (right cerebellar area VI), the left frontoparietal network (left opercular part of the inferior frontal gyrus), the salience network (bilateral insula, right triangular part of the inferior frontal gyrus, and left parahippocampal gyrus), and the visual network (left calcarine cortex and right cuneus) ($p < 0.005$, uncorrected).

Additionally, compared to healthy subjects, patients with schizophrenia exhibited significantly decreased functional connectivity within the basal ganglia network (right thalamus), the sensorimotor network I (right precentral gyrus and left central operculum), and the salience network (right medial and paracingulate gyri) ($p < 0.005$, uncorrected).

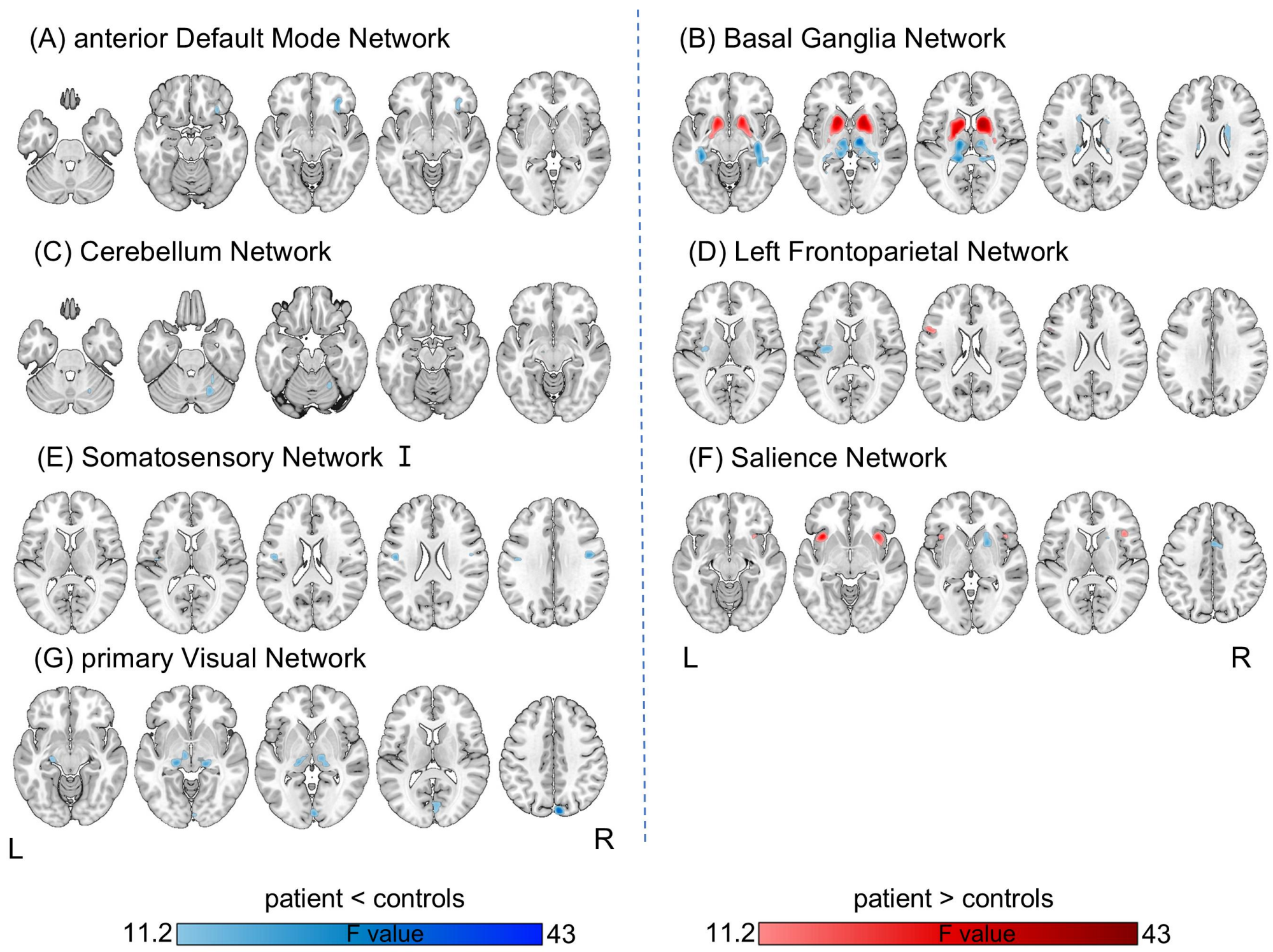

Figure S3. The main effects of group on intrinsic functional connectivity of rest state networks. ($p < 0.005$, uncorrected) *$p < 0.05$; **$p < 0.01$; ***$p < 0.001$

S4. Based on the main effect of sex from the ANOVA results, female participants exhibited significantly stronger functional connectivity within multiple networks compared to males, including the anterior default mode network (right orbital inferior frontal gyrus), the auditory network (bilateral superior temporal gyrus and right temporal pole), the basal ganglia network (right globus pallidus), the cerebellar network (left cerebellar area VI), the dorsolateral attention network (left insula), the left frontoparietal network (left orbital superior frontal gyrus and left angular gyrus), the posterior default mode network (left precuneus and left posterior cingulate cortex), the right frontoparietal network (right inferior parietal angular gyrus and right supramarginal gyrus), sensorimotor network I (bilateral precentral gyrus and left rolandic operculum), sensorimotor network II (median cingulate and paracingulate gyri and right gyrus rectus), the salience network (anterior cingulate and paracingulate gyri), and the visual network (left calcarine cortex and right lingual gyrus) ($p < 0.005$, uncorrected).

In contrast, female participants showed significantly weaker functional connectivity within the dorsolateral attention network (left precuneus) compared to males ($p < 0.005$, uncorrected).

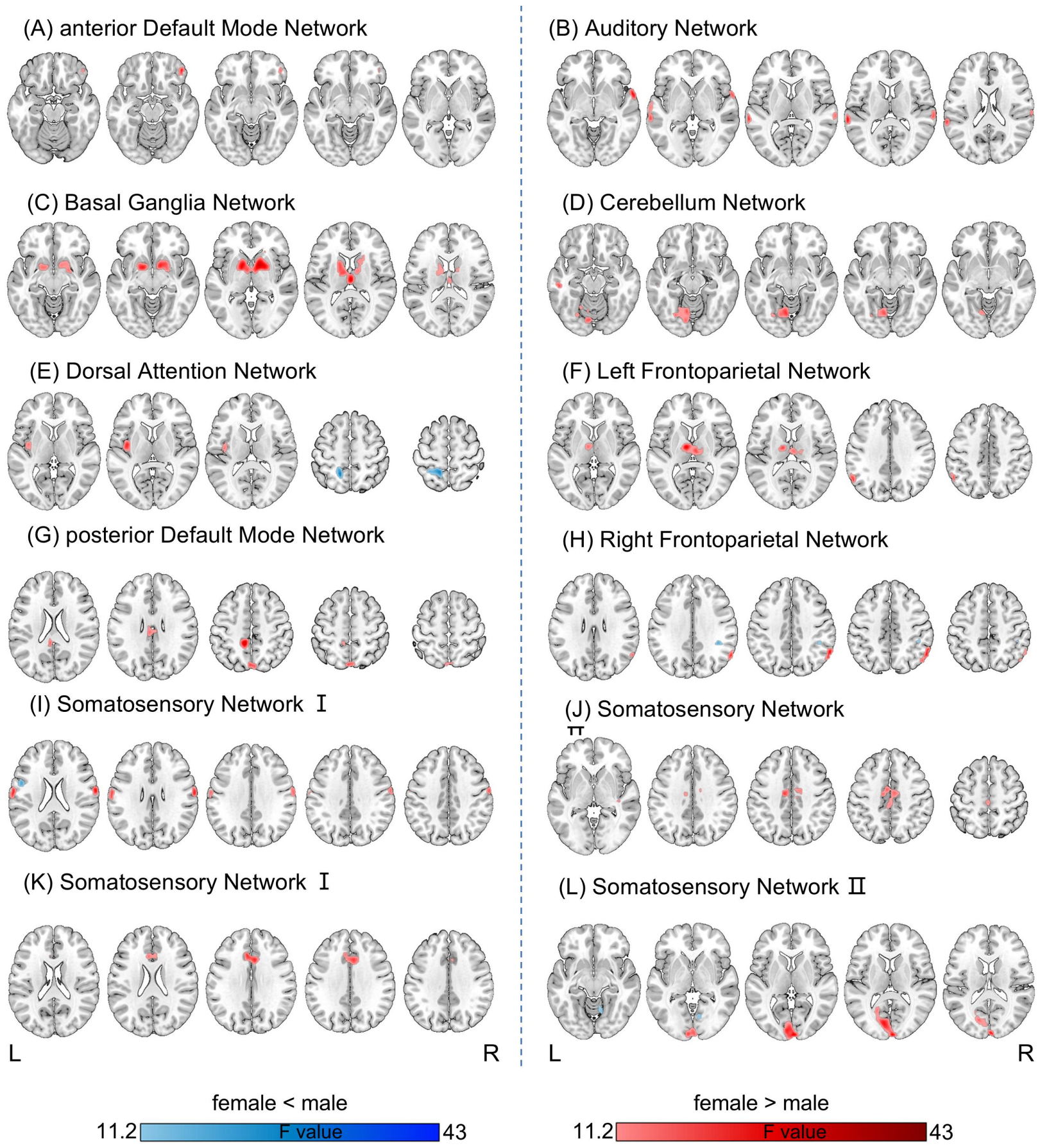

Figure S4. The main effects of sex on intrinsic functional connectivity of rest state networks. ($p < 0.005$, uncorrected) *$p < 0.05$; **$p < 0.01$; ***$p < 0.001$
